# Novel Homozygous Missense Variant in *GJA3* Connexin Domain Causing Congenital Nuclear and Cortical Cataracts

**DOI:** 10.3390/ijms23010240

**Published:** 2021-12-27

**Authors:** Abdullah Y. Hassan, Sairah Yousaf, Moran R. Levin, Osamah J. Saeedi, Saima Riazuddin, Janet L. Alexander, Zubair M. Ahmed

**Affiliations:** 1 Department of Otorhinolaryngology Head and Neck Surgery, School of Medicine, University of Maryland, Baltimore, MD 21201, USA; ayhassan126@gmail.com (A.Y.H.); sairayousaf61@gmail.com (S.Y.); sriazuddin@som.umaryland.edu (S.R.); 2 Department of Ophthalmology and Visual Sciences, School of Medicine, University of Maryland, Baltimore, MD 21201, USA; RLevin@som.umaryland.edu (M.R.L.); OSaeedi@som.umaryland.edu (O.J.S.); 3 Department of Molecular Biology and Biochemistry, School of Medicine, University of Maryland, Baltimore, MD 21201, USA

**Keywords:** *GJA3*, congenital cataract, African American, exome sequencing, nuclear cataract, cortical cataract

## Abstract

Congenital cataracts (CC) are responsible for approximately one-tenth of childhood blindness cases globally. Here, we report an African American family with a recessively inherited form of CC. The proband demonstrated decreased visual acuity and bilateral cataracts, with nuclear and cortical cataracts in the right and left eye, respectively. Exome sequencing revealed a novel homozygous variant (c.563A > G; p.(Asn188Ser)) in *GJA3*, which was predicted to be pathogenic by structural analysis. Dominantly inherited variants in *GJA3* are known to cause numerous types of cataracts in various populations. Our study represents the second case of recessive *GJA3* allele, and the first report in African Americans. These results validate *GJA3* as a bona fide gene for recessively inherited CC in humans.

## 1. Introduction

Congenital cataract (CC) is a clinically heterogenous condition that leads to imperviousness of the crystalline lens [1]. Cataracts accounts for approximately 10% of childhood vision impairment and blindness across the world, and can be subdivided according to the anatomical location within the lens and appearance, but are also broadly categorized by etiology [2]. Among genetic, traumatic, metabolic and infectious aspects, hereditary cataracts account for 22.3% of childhood cases worldwide [3]. The prevalence of CC is estimated to be 1 to 15 per 10,000 children globally [4]. Cataracts in infancy and early childhood can also impact visual development due to amblyopia [5]. Though pathogenesis of CC can be caused by multiple factors, genetic factors are the most common, with a predominance of autosomal dominant inheritance [6]. 

Cataract could manifest as an isolated ocular disease and/or as a component of complex syndromic disorders [7] such as Lowe oculocerebrorenal syndrome, a rare X-linked disorder, with characteristic features of intellectual disability, CC and later onset, renal dysfunction [8]. Cataracts as part of other syndromes, e.g., Hereditary hyperferritinemia-cataract syndrome (HHCS), display variation in onset and severity. The morphological features of cataracts in HHCS include numerous white breadcrumb-like opacities, abundant in the lens cortex [9]. In CC, the lens microarchitecture is disrupted and vacuoles form, which cause light scattering due to variability in the lens’s opaque density [10]. Over 100 genes have been reported to be involved in CC [7]. 

Approximately 25% of genetic mutations causative of CCs involve mutations in connexin genes [11]. The lens expresses three distinct connexins, connexin 43 (Cx43), Cx46, and Cx50, which oligomerize to form gap junctions. The avascular lens requires an extensive intercellular communication system using gap junctions to maintain tissue homeostasis, namely crystallin transparency and solubility [12]. Of three connexins, variants in Cx46 (*GJA3*) and Cx50 (*GJA8*) cause disruptions in hemichannel formation, the most common cause of autosomal dominant CC. Structurally impaired gap junctions due to genetic mutations disrupt hemichannel function and affect lens homeostasis, which ultimately alters the orderly arrangement of crystallin and causes cataract formation [13]. Recently, Cx46 is reported to play a key role in H_2_O_2_-induced apoptosis in human lens epithelial cells [14]. Predominantly heterozygous variants of *GJA3*, apart from one allele, are reported in individuals with congenital cataracts [15,16]. Here, we report a novel recessively inherited variant of *GJA3* in an African American family.

## 2. Results

### 2.1. Clinical Findings

**Figure 1:**
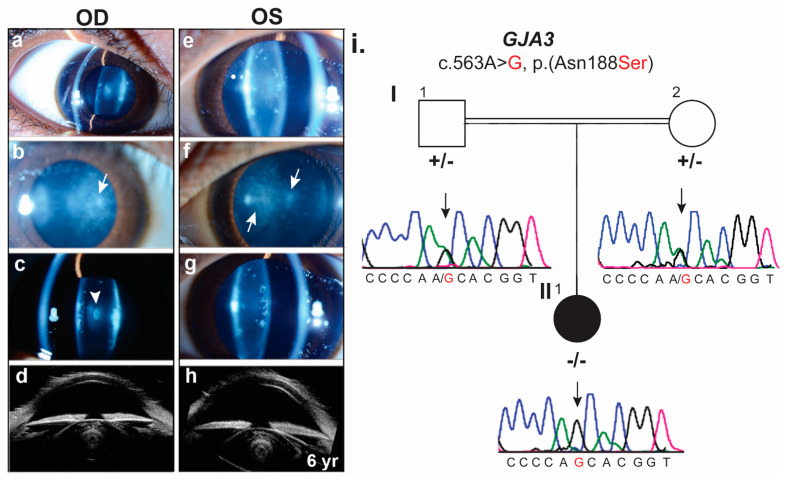
Clinical phenotype of proband affected by juvenile bilateral cataracts. (**a**–**h**) Slit lamp photographs of the OD (right) and OS (left) eye (**a**–**g**), respectively, demonstrate nuclear and cortical cataracts with dense fleck-like deposits in the anterior and posterior subcapsular regions, and diffuse “dust-like” pulverulent opacities (arrows). OD shows a small white central nuclear opacity (arrowhead; photographs courtesy of William Buie). Ultrasound images of OD and OS are shown in panels d and h, respectively. (**i**). Family of proband with CC showing segregation of *GJA3* missense variant. The affected individual is shown by a filled symbol. Sanger sequencing DNA chromatograms of *GJA3* for the heterozygous normal parents (I:1, I:2), and affected individual (II:1) are shown on the right side.

The proband (II: 1, Figure 1) was an African American female diagnosed with juvenile onset bilateral cataracts at age 6 after failed vision screening. Parents of the proband were siblings. Siblings of the proband include three full and additional half siblings, none of whom have cataracts. The proband’s medical history was significant due to depressive disorder, attention deficit hyperactivity disorder, and eczema. Her physical growth was normal for age in height but well below average for weight with weight Z-scores of −3 to −3.5, below the first percentile based on Centers for Disease Control and Prevention (Girls, 2–20 years) data. Prior ocular history was significant only for high myopia diagnosed at age 5. Her presenting best corrected visual acuity was 20/100 in the right and 20/150 in the left eye. Her cycloplegic refraction was −7.50 + 1.00 × 180 and −9.00 + 3.00 × 180, in each eye, respectively. Her slit lamp examination demonstrated nuclear and cortical cataracts in the right and left eye, respectively, with dense fleck-like deposits in the anterior and posterior subcapsular regions, and diffuse “dust-like” pulverulent opacities (Figure 1a–h). At the time of cataract surgery, the proband was found to have small corneal diameters of 10 mm in each eye and increased axial lengths of 24.5 and 24.6 mm, in each eye, respectively. Ultrasound biomicroscopy was performed at the time of cataract surgery (Figure 1a–h). 

### 2.2. Mutation Detection in GJA3

**Figure 2:**
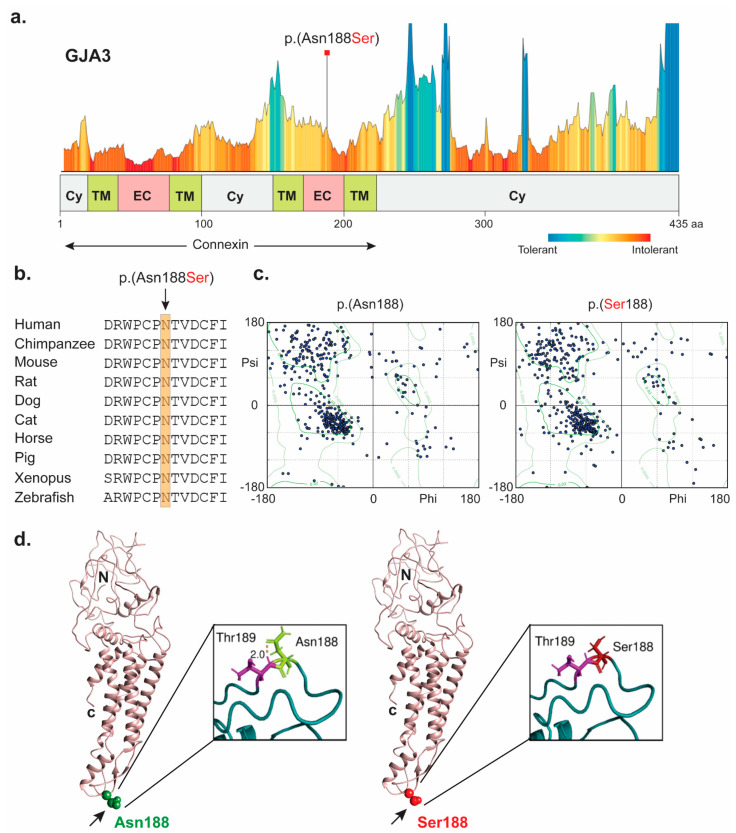
Computational analysis of human *GJA3* missense variant identified in the proband. (**a**) Tolerance and metadome landscape representation of *GJA3* with protein domains underneath. Connexin domain is marked with arrows. Missense variant (p.Asn188Ser) identified in the study is highlighted in red and found to be present in intolerant region. p.Asn188 is predicted to be present in extracellular region of connexin domain. (**b**) Amino acids conservation in orthologous species for the p.(Asn188Ser) variant. The wild type residue (p.Asn188) is conserved across a wide variety of species. (**c**). Shown are the Ramachandran plots of the wild type (left) and mutant (right) proteins PDB structures generated by Phyre2 and visualized by Chimera. Favored and allowed regions are shown in light color with two respective boundaries. The favored regions (inner boundary line) represent conformations with no steric clashes. (**d**) 3D structural modeling in Chimera of the *GJA3* protein, with the wild type and mutant residues shown, in ribbon and electrostatic potential representation, respectively. Asparagine and serine both are polar, non-charged (neutral) residues shown in white. However, blue and red colors represent amino acids with positive and negative charge, respectively. Abbreviations: Cy: cytoplasm; TM: transmembrane domain; EC: extracellular; C: c terminus; and N: n terminus.

Bioinformatics analysis of WES data generated using the DNA sample of proband revealed a homozygous variant (c.563A > G, p.(Asn188Ser)) in *GJA3*, which segregated with the phenotype in a recessive pattern (Figure 1i). The identified missense variant (p.(Asn188Ser)) is predicted to be the part of intolerant region (Figure 2a), deleterious (Supplementary Table S1), and replaces evolutionary conserved residue (Figure 2b). The p.Asn188 is located in the second extracellular loop (E2) of the protein (Figure 2d), a domain critical for the activity of the protein [12,13]. The mutant residue (serine) is predicted to be small in size with loss of interactions, due to the lack of carbonyl group (as in asparagine), which interacts with the adjacent residues. The phi angle with the residue of interest shifted further outside of the permitted region of the Ramachandran plot (Figure 2c), further enforcing the fact that the loss of this stabilizing interaction affects the protein structure.

## 3. Discussion

The major CC-associated proteins include transcription factors, crystallin, structural proteins, and membrane proteins (connexins) [14]. The lens expresses three distinct connexins—Cx43, Cx46, and Cx50—all of which appear to have different functions in maintaining lens homeostasis [8]. Extracellular domains of connexins play a key role in both mediating hemichannel docking [15,16] and regulating voltage gating of the channel [17]. Gap junctions within the lens help maintain an environment that favors crystallin solubility and fiber transparency by coupling the metabolically active epithelium and the organelle lacking lens fibers into a syncytium.

In human, *GJA3* (Cx43) has been associated with a variety of inherited forms of CC (Table 1), though the most common remains zonular cataracts [12]. In our study, parents of the proband were siblings and carriers of the same variant of *GJA3*. Her clinical presentation showed bilateral cataracts upon slit lamp examination, with decreased corneal diameter and increased axial length of both eyes. There is a high proportion of genetically inherited forms of cataract with a substantial heterogeneity in both genetics and phenotype. Therefore, the arising phenotype could be the consequence of pleiotropic effect of lens proteins, which would thus show significant inter and intra-familial inconsistencies. 

As of August 2021, forty-nine different cataract-associated variants in *GJA3* have been identified according to the Human Gene Mutation Database, and almost all of them cause cataract in a dominant fashion (Table 1; Supplementary Table S2). The only exception was the c.950dupG (p.(His318Profs*8) allele, which segregated in a recessive fashion in a Pakistani family [18]. Our study presents a second case of CC with a homozygous variant of *GJA3* (Table 1). The clinical phenotype of the previously reported affected individuals with p.(His318Profs*8) homozygous variant include bilateral nuclear cataract, and secondary glaucoma after cataract surgery [18]. Similarly, the affected proband in our family also has nuclear cataract. She subsequently underwent uncomplicated cataract surgery with IOL implantation in both eyes, followed by yttrium aluminum garnet (YAG) laser capsulotomy in both eyes at cataract post-operative year 2. Following YAG capsulotomy, her visual acuity improved to 20/20 in each eye and has remained at this level at the most recent follow up post-operative year 4 (age 10).

Our study also represents the first reported case of *GJA3* in an individual of African American ancestry (Table 1). Two studies previously reported heterozygous missense variants (p.(Asn188Thr), p.(Asn188Ile), present in second extracellular loop) in Chinese families [10,19], impacting the same *GJA3* residue that is mutated in our case. However, in all three scenarios, the substituted amino acids are different (Table 1). The major clinical features of the reported dominant alleles (p.(Asn188Thr), p.(Asn188Ile)) include congenital nuclear pulverulent cataracts, dense coral-like opacities in nuclear region of the lens surrounded by blue dust-like opacities in the cortical zone [10,19]. However, in our case, the obligated carriers of the p.(Asn188Ser) variant were examined and had no cataracts or other eye problems. The second extracellular loop helps *GJA3* anchor in hemichannel gap junctions. The p.Asn188Thr has been reported to have no impact on electrical assets of xenopus hemichannels. However, Asn188 is crucial for hemichannel docking by forming hydrogen bonds with amino acids arginine, threonine, and aspartic acid at positions 180, 189 and 191, respectively. Thus, it is reported that Asn188 replacement with threonine obstructs the anchorage of connexons to form gap junction channels [20]. However, it is worth mentioning that different alleles of *GJA3* cause different phenotypes at different locations within the lens. It is possible that the genetic background modifies the resulting phenotype of *GJA3* mutations, though differences between the alleles may directly account for the different outcomes. 

PVS:1: Null variant (nonsense, frameshift, canonical ±1 or 2 splice sites, initiation codon, single or multiexon deletion) in a gene where LOF is a known mechanism of disease (Pathogenic, Very Strong).

PS1: Same amino acid change as a previously established pathogenic variant regardless of nucleotide change (Pathogenic, Strong).

PM1: Located in a mutational hot spot and/or critical and well-established functional domain (e.g., active site of an enzyme) without benign variation (Pathogenic, Moderate).

PM2: Absent from controls (or at extremely low frequency if recessive) in Exome Sequencing Project, 1000 Genomes Project, or Exome Aggregation Consortium (Pathogenic, Moderate).

PM5: Novel missense change at an amino acid residue where a different missense change determined to be pathogenic has been seen before (Pathogenic, Moderate).

PP2: Missense variant in a gene that has a low rate of benign missense variation and in which missense variants are a common mechanism of disease (Pathogenic, Supporting).

PP3: Multiple lines of computational evidence support a deleterious effect on the gene or gene product (conservation, evolutionary, splicing impact, etc.) (Pathogenic, Supporting).

PP5: Reputable source recently reports variant as pathogenic, but the evidence is not available to the laboratory to perform an independent evaluation (Pathogenic, Supporting).

BS1: Allele frequency is greater than expected for disorder (Benign, Strong).

BS2: Observed in a healthy adult individual for a recessive (homozygous), dominant (heterozygous), or X-linked (hemizygous) disorder, with full penetrance expected at an early age (Benign, Strong).

BP4: Multiple lines of computational evidence suggest no impact on gene or gene product (conservation, evolutionary, splicing impact, etc.) (Benign, Supporting).

BP6: Reputable source recently reports variant as benign, but the evidence is not available to the laboratory to perform an independent evaluation (Benign, Supporting).

BP7: A synonymous (silent) variant for which splicing prediction algorithms predict no impact to the splice consensus sequence nor the creation of a new splice site AND the nucleotide is not highly conserved (Benign, Supporting).

## 4. Materials and Methods

### 4.1. Clinical Evaluation 

Visual acuity was assessed by using the standard Snellen chart. Fundoscopy and slit lamp biomicroscopy were also performed. Axial length measurement and ultrasound biomicroscopy imaging were performed using the Aviso Ultrasound Platform A/B UBM with a 50 MHz linear transducer (Quantel Medical, Clermont-Ferrand, France). Blood samples were collected from all the participants for DNA extraction. 

### 4.2. Exome Sequencing and Bioinformatic Analyses

Whole exome sequencing (WES) was performed on the proband DNA sample, and data were filtered using the criteria previously described [11]. Sanger sequencing was used for the segregation analysis of identified variants in the family. In silico analysis and three-dimensional (3D) molecular modeling were performed using various programs (see web resources). Finally, the Varsome.com online tool was used for the American College of Medical Genetics and Genomics classification of the *GJA3* variants.

## Figures and Tables

**Table 1:** *GJA3* variants, inheritance, cataract type, ethnicity or origin, and ACMG classification.

DNA Change	Amino Acid Change	Zygosity	Cataract Type	Ethnicity/Location
c.-17-22C > G or c.-39C > G	-	Heterozygous	Nuclear	Chinese
c.1A > G	p.(Met1Val)	Heterozygous	Congenital	Indian
c.5G > A	p.(Gly2Asp)	Heterozygous	Nuclear pulverulent and posterior polar	Chinese
c.7G > C	p.(Asp3His)	Heterozygous	Congenital	Caucasian, Middle Eastern, Asian
c.7G > T	p.(Asp3Tyr)	Heterozygous	Zonular pulverulent	Hispanic
c.32T > C	p.(Leu11Ser)	Heterozygous	Ant-egg cataract	Danish
c.56C > T	p.(Thr19Met)	Heterozygous	Posterior-polar, nuclear-lamellar	Indian, Southeastern Australia
c.64G > A	p.(Gly22Ser)	Heterozygous	Pulverized cataract	Chinese
c.82G > T	p.(Val28Leu)	Heterozygous	Congenital	British
c.82G > A	p.(Val28Met)	Heterozygous	Posterior cortical and anterior capsular	Indian
c.84G > A	p.(Val28Val)	Heterozygous	Congenital, posterior subcapsular cataract, nystagmus	Indian
c.92T > A	p.(Ile31Asn)	Heterozygous	Bilateral microphthalmia, microcornea, and membranous cataract	Indian
c.96C > A	p.(Phe32Leu)	Heterozygous	Congenital nuclear pulverulent	Chinese
c.98G > T	p.(Arg33Leu)	Heterozygous	Congenital	Indian
c.125A > C	p.(Glu42Ala)	Heterozygous	pulverulent	Chinese
c.130G > A	p.(Val44Met)	Heterozygous	Central nuclear cataract with punctate cortical opacities	Chinese, Caucasian, Middle Eastern and Asian
c.134G > C	p.(Trp45Ser)	Heterozygous	Congenital nuclear cataract	Chinese
c.139G > A	p.(Asp47Asn)	Heterozygous	Congenital nuclear cataract	Chinese
c.143A > G	p.(Glu48Gly)	Heterozygous	Congenital	Chinese
c.148T > C	p.(Ser50Pro)	Heterozygous	Congenital	European, Chinese
c.163A > G	p.(Asn55Asp)	Heterozygous	Congenital	Chinese
c.176C > T	p.(Pro59Leu)	Heterozygous	Nuclear punctate, Congenital	Caucasian, Middle Eastern and Asian, Chinese
c.184G > A	p.(Glu62Lys)	Heterozygous	Congenital	Caucasian
c.188A > G	p.(Asn63Ser)	Heterozygous	Congenital Zonular Pulverulent	Caucasian
c.199G > C	p.(Asp67His)	Heterozygous	Cataract	Chinese
c.226C > G	p.(Arg76Gly)	Heterozygous	Cataract	Indian
c.227G > A	p.(Arg76His)	Heterozygous	Congenital with incomplete penetrance	Australian
c.260C > T	p.(Thr87Met)	Heterozygous	Peal box cataract	Indian
c.268C > T	p.(Leu90Phe)	Heterozygous	Nuclear	Chinese
c.415G > A	p.(Val139Met)	Heterozygous	Nuclear	Chinese
c.427G > A	p.(Gly143Arg)	Heterozygous	Congenital Coppock-like cataract	Chinese
c.428G > A	p.(Gly143Glu)	Heterozygous	Congenital nuclear cataract	Chinese
c.443C > T	p.(Thr148Ile)	Heterozygous	Bilateral pulverulent nuclear	Chinese
c.466A > C	p.(Lys156Gln)	Heterozygous	-	Southeastern Australia
c.559C > T	p.(Pro187Ser)	Heterozygous	Congenital, central nuclear opacity	Chinese
c.560C > T	p.(Pro187Leu)	Heterozygous	Congenital zonular pulverulent cataract	Caucasian
c.563A > T	p.(Asn188Ile)	Heterozygous	Congenital nuclear coralliform cataracts	Chinese
c.563A > C	p.(Asn188Thr)	Heterozygous	congenital nuclear pulverulent cataract	Chinese
**c.563A > G**	**p.(Asn188Ser)**	**Homozygous**	**Juvenile onset, nuclear**	**African American**
c.578T > C	p.(Phe193Ser)	Heterozygous	Syndromic	Caucasian
c.589C > T	p.(Pro197Ser)	Heterozygous	Congenital	Indian
c.596A > C	p.(Glu199Ala)	Heterozygous	Congenital	European
c.616T > A	p.(Phe206Ile)	Heterozygous	Congenital, nuclear	Chinese
c.771dupC	p.(Ser258Glnfs*68)	Heterozygous	Isolated lamellar cataract	British
**c.950dupG**	p.(His318Profs*8)	**Homozygous**	**Nuclear, secondary glaucoma after cataract surgery**	**Pakistani**
c.1137dupC	p.(Ser380Glnfs*88)	Heterozygous	Congenital Zonular Pulverulent 3	Caucasian
c.1152dupG	p.(Ser385Glufs*83)	Heterozygous	Cataract 14	Chinese
c.1189dupG	p.(Ala397Glyfs*71)	Heterozygous	Congenital coralliform cataract	Chinese
c.1200dupC	p.(Ala401Argfs*67)	Heterozygous	Nuclear	Chinese
c.1143_1165del23	p.(Ser381Argfs*79)	Heterozygous	Congenital	Chinese

* American College of Medical Genetics and Genomics classifications are given based on the https://Varsome.com (accessed on 15 November 2021) program predictions. Given in bold are the two known recessively inherited variants of *GJA3*.

## Data Availability

The variant has been submitted to the ClinVar database: Accession number: SCV001787094.

## Supplementary Materials

The following are available online at https://www.mdpi.com/article/10.3390/ijms23010240/s1.
