# Supplementary material for: Novel Homozygous Missense Variant in GJA3 Connexin Domain Causing Congenital Nuclear and Cortical Cataracts
Source: Int J Mol Sci. 2021 Dec 27;23(1):240. doi: 10.3390/ijms23010240 (PMC8745576; doi:10.3390/ijms23010240)
Supplement: Supplementary file 1 [file ijms-23-00240-s001.zip › ijms-1488006-supplementary.pdf]

Supplementary Table S1. Homozygous recessive variant of *GJA3* causing congenital cataracts

	GJA3
hg19 Position	chr13:20716865
Genomic region	13q12.11
Reference genomic allele	T
Alternate genomic allele	C
GenBank	NM_021954.4
cDNA change	c.563A>G
Amino acid change	p.(Asn188Ser)
Segregates with the phenotype	Yes
dbSNP ID	rs140332366
ExAC allele frequency	0.000003982
ExAC Europeans (Non-Finnish) MAF	0.000008813
1000 genome	Absent
ESP-6500 allele frequency	0.000077
ESP-6500 European American MAF	0.000116
ESP-6500 African American MAF	Absent
ClinVar*	Pathogenic
SIFT	Damaging
Polyphen2	Probably damaging
MutationAssessor	Medium
Fathmm	Damaging
Provean	Deleterious
CADD	25.1

GJA3 encodes Gap junction Alpha 3 protein

Supplementary Table S2. *GJA3* variants, inheritance, cataract type, ethnicity or origin

DNA change	Amino acid change	dbSNP ID	SIFT	PolyPhen-2	Likelihood ratio test (LRT)	Fathmm	CADD	gnomAD MEF*	Reference
c.-17-22C>G or c.-39C>G	-	*rs1445611624*	-	-	-	-	-	$2.737x10^{-5}$	(1)
c.1A>G	p.(Met1Val)	-	Damaging	Probably damaging	Deleterious	Damaging	23.5	0	(2)
c.5G>A	p.(Gly2Asp)	*rs397514703*	Damaging	Probably damaging	Deleterious	Damaging	26.6	0	(3)
c.7G>C	p.(Asp3His)	*rs864309694*	Damaging	Probably damaging	Deleterious	Damaging	26	0	(4)
c.7G>T	p.(Asp3Tyr)	-	Damaging	Probably damaging	Deleterious	Damaging	27.4	0	(5), (6), (7)
c.32T>C	p.(Leu11Ser)	-	Damaging	Probably damaging	Neutral	Damaging	22.5	0	(8), (7)
c.56C>T	p.(Thr19Met)	*rs1114167307*	Damaging	Probably damaging	Deleterious	Damaging	29	0	(9), (10), (11)
c.64G>A	p.(Gly22Ser)	-	Damaging	Probably damaging	Deleterious	Damaging	33	0	(12)
c.82G>T	p.(Val28Leu)	-	Damaging	Probably damaging	Deleterious	Damaging	32	0	(13)
c.82G>A	p.(Val28Met)	-	Damaging	Probably damaging	Deleterious	Damaging	31	0	(14)
c.84G>A	p.(Val28Val)	-	-	-	-	-	-	$6.552x10^{-4}$	(15)
c.92T>A	p.(Ile31Asn)	-	Damaging	Probably damaging	Deleterious	Damaging	25.6	0	(16)
c.96C>A	p.(Phe32Leu)	-	Damaging	Probably damaging	Deleterious	Damaging	27.2	0	(17)
c.98G>T	p.(Arg33Leu)	-	Damaging	Probably damaging	Deleterious	Damaging	33	0	(18)
c.125A>C	p.(Glu42Ala)	-	Damaging	Probably damaging	Deleterious	Damaging	24.1	0	(19)
c.130G>A	p.(Val44Met)	-	Damaging	Probably damaging	Deleterious	Damaging	26.1	$4.062x10^{-6}$	(20), (4), (21)
c.134G>C	p.(Trp45Ser)	-	Damaging	Probably damaging	Deleterious	Damaging	24.9	0	(22)
c.139G>A	p.(Asp47Asn)	-	Damaging	Probably damaging	Deleterious	Damaging	25.8	0	(23)
c.143A>G	p.(Glu48Gly)	-	Damaging	Probably damaging	Deleterious	Damaging	24.1	0	(24)
c.148T>C	p.(Ser50Pro)	-	Damaging	Probably damaging	Deleterious	Damaging	22.9	0	(25), (26)
c.163A>G	p.(Asn55Asp)	-	Tolerated	Possibly damaging	Deleterious	Damaging	22.9	0	(27)
c.176C>T	p.(Pro59Leu)	*rs864309691*	Damaging	Probably damaging	Deleterious	Damaging	29.7	0	(28), (4), (29)

c.184G>A	p.(Glu62Lys)	-	Damaging	Benign	Deleterious	Damaging	33	0	(25)
c.188A>G	p.(Asn63Ser)	*rs121917823*	Damaging	Probably damaging	Deleterious	Damaging	24	0	(30)
c.199G>C	p.(Asp67His)	-	Damaging	Probably damaging	Deleterious	Damaging	27.8	0	(31)
c.226C>G	p.(Arg76Gly)	-	Damaging	Probably damaging	Deleterious	Damaging	29	0	(14)
c.227G>A	p.(Arg76His)	*rs121917827*	Damaging	Probably damaging	Deleterious	Damaging	34	0	(32)
c.260C>T	p.(Thr87Met)	*rs864309687*	Damaging	Probably damaging	Deleterious	Damaging	28.7	0	(18), (4)
c.268C>T	p.(Leu90Phe)	-	Damaging	Probably damaging	Deleterious	Damaging	26.5	0	(19)
c.415G>A	p.(Val139Met)	-	Damaging	Benign	Neutral	Damaging	22.8	0	(1)
c.427G>A	p.(Gly143Arg)	*rs398122937*	Damaging	Probably damaging	Deleterious	Damaging	25.7	0	(33), (34), (35)
c.428G>A	p.(Gly143Glu)	-	Damaging	Probably damaging	Deleterious	Damaging	24.9	0	(36)
c.443C>T	p.(Thr148Ile)	-	Damaging	Probably damaging	Deleterious	Damaging	25.7	0	(37)
c.466A>C	p.(Lys156Gln)	*rs1114167308*	Damaging	Probably damaging	Deleterious	Damaging	25.2	0	(11)
c.559C>T	p.(Pro187Ser)	-	Damaging	Probably damaging	Deleterious	Damaging	26.5	0	(38)
c.560C>T	p.(Pro187Leu)	*rs121917825*	Damaging	Probably damaging	Deleterious	Damaging	27.1	0	(39)
c.563A>T	p.(Asn188Ile)	*rs140332366*	Damaging	Probably damaging	Deleterious	Damaging	27.2	0	(33)
c.563A>C	p.(Asn188Thr)	*rs140332366*	Damaging	Probably damaging	Deleterious	Damaging	25.8	0	(40), (41)
**c.563A>G**	**p.(Asn188Ser)**	***rs140332366***	**Damaging**	**Probably damaging**	**Deleterious**	**Damaging**	**25.1**	**$3.982x10^{-5}$**	**This study**
c.578T>C	p.(Phe193Ser)	-	Damaging	Probably damaging	Deleterious	Damaging	28.8	0	(25)
c.589C>T	p.(Pro197Ser)	-	Damaging	Probably damaging	Deleterious	Damaging	26.9	0	(15)
c.596A>C	p.(Glu199Ala)	-	Damaging	Probably damaging	Deleterious	Damaging	26.6	0	(25)
c.616T>A	p.(Phe206Ile)	*rs397514704*	Damaging	Probably damaging	Deleterious	Damaging	27.8	0	(42)
c.771dupC	p.(Ser258Glnfs*68)	*rs752926638*	-	-	-	-	-	0	(13)
**c.950dupG**	**p.(His318Profs*8)**	**-**	-	-	-	-	-	**0**	**(43)**
c.1137dupC	p.(Ser380Glnfs*88)	-	-	-	-	-	-	0	(30)
c.1152dupG	p.(Ser385Glufs*83)	-	-	-	-	-	-	0	(44)

c.1189dupG	p.(Ala397Glyfs*71)	-	-	-	-	-	-	0	(45)
c.1200dupC	p.(Ala401Argfs*67)	-	-	-	-	-	-	0	(46)
c.1143_1165del23	p.(Ser381Argfs*79)	-	-	-	-	-	-	0	(47)

Supplementary References:

1. Zhou Z, Wang B, Hu S, Zhang C, Ma X, Qi Y. Genetic variations in GJA3, GJA8, LIM2, and age-related cataract in the Chinese population: a mutation screening study. Mol Vis. 2011;17:621-6.
2. Kumar M, Agarwal T, Kaur P, Kumar M, Khokhar S, Dada R. Molecular and structural analysis of genetic variations in congenital cataract. Mol Vis. 2013;19:2436-50.
3. Yao K, Wang W, Zhu Y, Jin C, Shentu X, Jiang J, et al. A novel GJA3 mutation associated with congenital nuclear pulverulent and posterior polar cataract in a Chinese family. Hum Mutat. 2011;32(12):1367-70.
4. Ma AS, Grigg JR, Ho G, Prokudin I, Farnsworth E, Holman K, et al. Sporadic and Familial Congenital Cataracts: Mutational Spectrum and New Diagnoses Using Next-Generation Sequencing. Hum Mutat. 2016;37(4):371-84.
5. Addison PK, Berry V, Holden KR, Espinal D, Rivera B, Su H, et al. A novel mutation in the connexin 46 gene (GJA3) causes autosomal dominant zonular pulverulent cataract in a Hispanic family. Mol Vis. 2006;12:791-5.
6. Schlingmann B, Schadzek P, Busko S, Heisterkamp A, Ngezahayo A. Cataract-associated D3Y mutation of human connexin46 (hCx46) increases the dye coupling of gap junction channels and suppresses the voltage sensitivity of hemichannels. J Bioenerg Biomembr. 2012;44(5):607-14.
7. Tong JJ, Sohn BC, Lam A, Walters DE, Vertel BM, Ebihara L. Properties of two cataract-associated mutations located in the NH2 terminus of connexin 46. Am J Physiol Cell Physiol. 2013;304(9):C823-32.
8. Hansen L, Yao W, Eiberg H, Funding M, Riise R, Kjaer KW, et al. The congenital "ant-egg" cataract phenotype is caused by a missense mutation in connexin46. Mol Vis. 2006;12:1033-9.
9. Santhiya ST, Kumar GS, Sudhakar P, Gupta N, Klopp N, Illig T, et al. Molecular analysis of cataract families in India: new mutations in the CRYBB2 and GJA3 genes and rare polymorphisms. Mol Vis. 2010;16:1837-47.
10. Tong JJ, Minogue PJ, Kobeszko M, Beyer EC, Berthoud VM, Ebihara L. The connexin46 mutant, Cx46T19M, causes loss of gap junction function and alters hemi-channel gating. J Membr Biol. 2015;248(1):145-55.
11. Javadiyan S, Craig JE, Souzeau E, Sharma S, Lower KM, Mackey DA, et al. High-Throughput Genetic Screening of 51 Pediatric Cataract Genes Identifies Causative Mutations in Inherited Pediatric Cataract in South Eastern Australia. G3 (Bethesda). 2017;7(10):3257-68.
12. Ye Y, Wu M, Qiao Y, Xie T, Yu Y, Yao K. Identification and preliminary functional analysis of two novel congenital cataract associated mutations of Cx46 and Cx50. Ophthalmic Genet. 2019;40(5):428-35.
13. Berry V, Ionides A, Pontikos N, Moghul I, Moore AT, Quinlan RA, et al. Whole Exome Sequencing Reveals Novel and Recurrent Disease-Causing Variants in Lens Specific Gap Junctional Protein Encoding Genes Causing Congenital Cataract. Genes (Basel). 2020;11(5).
14. Devi RR, Reena C, Vijayalakshmi P. Novel mutations in GJA3 associated with autosomal dominant congenital cataract in the Indian population. Mol Vis. 2005;11:846-52.
15. Ponnam SP, Ramesha K, Matalia J, Tejwani S, Ramamurthy B, Kannabiran C. Mutational screening of Indian families with hereditary congenital cataract. Mol Vis. 2013;19:1141-8.
16. Vidya NG, Rajkumar S, Vasavada AR. Genetic investigation of ocular developmental genes in 52 patients with anophthalmia/microphthalmia. Ophthalmic Genet. 2018;39(3):344-52.

17. Jiang H, Jin Y, Bu L, Zhang W, Liu J, Cui B, et al. A novel mutation in GJA3 (connexin46) for autosomal dominant congenital nuclear pulverulent cataract. Mol Vis. 2003;9:579-83.

18. Guleria K, Sperling K, Singh D, Varon R, Singh JR, Vanita V. A novel mutation in the connexin 46 (GJA3) gene associated with autosomal dominant congenital cataract in an Indian family. Mol Vis. 2007;13:1657-65.

19. Yang Z, Li Q, Ma X, Zhu SQ. Mutation analysis in Chinese families with autosomal dominant hereditary cataracts. Curr Eye Res. 2015;40(12):1225-31.

20. Zhou Z, Hu S, Wang B, Zhou N, Zhou S, Ma X, et al. Mutation analysis of congenital cataract in a Chinese family identified a novel missense mutation in the connexin 46 gene (GJA3). Mol Vis. 2010;16:713-9.

21. Chen L, Su D, Li S, Guan L, Shi C, Li D, et al. The connexin 46 mutant (V44M) impairs gap junction function causing congenital cataract. J Genet. 2017;96(6):969-76.

22. Ma ZW, Zheng JQ, Li J, Li XR, Tang X, Yuan XY, et al. Two novel mutations of connexin genes in Chinese families with autosomal dominant congenital nuclear cataract. Br J Ophthalmol. 2005;89(11):1535-7.

23. Yang G, Xing B, Liu G, Lu X, Jia X, Lu X, et al. A novel mutation in the GJA3 (connexin46) gene is associated with autosomal dominant congenital nuclear cataract in a Chinese family. Mol Vis. 2011;17:1070-3.

24. Li B, Liu Y, Liu Y, Guo H, Hu Z, Xia K, et al. Identification of a GJA3 Mutation in a Large Family with Bilateral Congenital Cataract. DNA Cell Biol. 2016;35(3):135-9.

25. Gillespie RL, O'Sullivan J, Ashworth J, Bhaskar S, Williams S, Biswas S, et al. Personalized diagnosis and management of congenital cataract by next-generation sequencing. Ophthalmology. 2014;121(11):2124-37 e1-2.

26. Li H, Jiang H, Rong R, Jiang J, Ji D, Song W, et al. Identification of GJA3 p.S50P Mutation in a Chinese Family with Autosomal Dominant Congenital Cataract and Its Underlying Pathogenesis. DNA Cell Biol. 2020;39(10):1760-6.

27. Hu Y, Gao L, Feng Y, Yang T, Huang S, Shao Z, et al. Identification of a novel mutation of the gene for gap junction protein alpha3 (GJA3) in a Chinese family with congenital cataract. Mol Biol Rep. 2014;41(7):4753-8.

28. Bennett TM, Mackay DS, Knopf HL, Shiels A. A novel missense mutation in the gene for gap-junction protein alpha3 (GJA3) associated with autosomal dominant "nuclear punctate" cataracts linked to chromosome 13q. Mol Vis. 2004;10:376-82.

29. Wang L, Chen Y, Chen X, Sun X. Further evidence for P59L mutation in GJA3 associated with autosomal dominant congenital cataract. Indian J Ophthalmol. 2016;64(7):508-12.

30. Mackay D, Ionides A, Kibar Z, Rouleau G, Berry V, Moore A, et al. Connexin46 mutations in autosomal dominant congenital cataract. Am J Hum Genet. 1999;64(5):1357-64.

31. Sun Y, Man J, Wan Y, Pan G, Du L, Li L, et al. Targeted next-generation sequencing as a comprehensive test for Mendelian diseases: a cohort diagnostic study. Sci Rep. 2018;8(1):11646.

32. Burdon KP, Wirth MG, Mackey DA, Russell-Eggitt IM, Craig JE, Elder JE, et al. A novel mutation in the Connexin 46 gene causes autosomal dominant congenital cataract with incomplete penetrance. J Med Genet. 2004;41(8):e106.

33. Zhang L, Qu X, Su S, Guan L, Liu P. A novel mutation in GJA3 associated with congenital Coppock-like cataract in a large Chinese family. Mol Vis. 2012;18:2114-8.

34. Ren Q, Riquelme MA, Xu J, Yan X, Nicholson BJ, Gu S, et al. Cataract-causing mutation of human connexin 46 impairs gap junction, but increases hemichannel function and cell death. PLoS One. 2013;8(9):e74732.

35. Hu Z, Riquelme MA, Wang B, Bugay V, Brenner R, Gu S, et al. Cataract-associated connexin 46 mutation alters its interaction with calmodulin and function of hemichannels. J Biol Chem. 2018;293(7):2573-85.

36. Yuan L, Guo Y, Yi J, Xiao J, Yuan J, Xiong W, et al. Identification of a novel GJA3 mutation in congenital nuclear cataract. Optom Vis Sci. 2015;92(3):337-42.

37. Yao Y, Zheng X, Ge X, Xiu Y, Zhang L, Fang W, et al. Identification of a novel GJA3 mutation in a large Chinese family with congenital cataract using targeted exome sequencing. PLoS One. 2017;12(9):e0184440.

38. Ding X, Wang B, Luo Y, Hu S, Zhou G, Zhou Z, et al. A novel mutation in the connexin 46 (GJA3) gene associated with congenital cataract in a Chinese pedigree. Mol Vis. 2011;17:1343-9.

39. Rees MI, Watts P, Fenton I, Clarke A, Snell RG, Owen MJ, et al. Further evidence of autosomal dominant congenital zonular pulverulent cataracts linked to 13q11 (CZP3) and a novel mutation in connexin 46 (GJA3). Hum Genet. 2000;106(2):206-9.

40. Li Y, Wang J, Dong B, Man H. A novel connexin46 (GJA3) mutation in autosomal dominant congenital nuclear pulverulent cataract. Mol Vis. 2004;10:668-71.

41. Schadzek P, Schlingmann B, Schaarschmidt F, Lindner J, Koval M, Heisterkamp A, et al. The cataract related mutation N188T in human connexin46 (hCx46) revealed a critical role for residue N188 in the docking process of gap junction channels. Biochim Biophys Acta. 2016;1858(1):57-66.

42. Wang KJ, Zhu SQ. A novel p.F206I mutation in Cx46 associated with autosomal dominant congenital cataract. Mol Vis. 2012;18:968-73.

43. Micheal S, Niewold ITG, Siddiqui SN, Zafar SN, Khan MI, Bergen AAB. Delineation of Novel Autosomal Recessive Mutation in GJA3 and Autosomal Dominant Mutations in GJA8 in Pakistani Congenital Cataract Families. Genes (Basel). 2018;9(2).

44. Li S, Zhang J, Cao Y, You Y, Zhao X. Novel mutations identified in Chinese families with autosomal dominant congenital cataracts by targeted next-generation sequencing. BMC Med Genet. 2019;20(1):196.

45. Zhou D, Ji H, Wei Z, Guo L, Li Y, Wang T, et al. A novel insertional mutation in the connexin 46 (gap junction alpha 3) gene associated with autosomal dominant congenital cataract in a Chinese family. Mol Vis. 2013;19:789-95.

46. Cui XK, Zhu KK, Zhou Z, Wan SM, Dong Y, Wang XC, et al. A novel frameshift mutation in CX46 associated with hereditary dominant cataracts in a Chinese family. Int J Ophthalmol. 2017;10(5):684-90.

47. Sun W, Xiao X, Li S, Guo X, Zhang Q. Mutation analysis of 12 genes in Chinese families with congenital cataracts. Mol Vis. 2011;17:2197-206.
